# Timing of Atrial Fibrillation Ablation and Outcomes in Elderly Patients with HFrEF: A Propensity-Matched Study

**DOI:** 10.3390/jcm15062191

**Published:** 2026-03-13

**Authors:** Ibrahim Mortada, Aaron W. Lee, Dalton Buckingham, George M. Bianco, Thomas A. Blackwell, Hani Jneid

**Affiliations:** 1 Department of Cardiovascular Medicine, University of Texas Medical Branch, Galveston, TX 77555, USA; 2 John Sealy School of Medicine, University of Texas Medical Branch, Galveston, TX 77555, USA; 3 Department of Internal Medicine, University of Texas Medical Branch, Galveston, TX 77555, USA

**Keywords:** atrial fibrillation, elderly patients, heart failure with reduced ejection fraction, timing of intervention, heart rate variability

## Abstract

**Background:** Catheter ablation is an established rhythm-control strategy for atrial fibrillation, yet the optimal timing of ablation in older patients with heart failure with reduced ejection fraction remains uncertain. **Methods:** We conducted a retrospective study using the TriNetX research network, including adults aged ≥ 60 years with atrial fibrillation and systolic heart failure who underwent catheter ablation between 2010 and 2022. Patients were classified by the timing of catheter ablation relative to cardiac diagnosis, with early ablation defined as occurring within 1 year and delayed ablation defined as occurring after 1 year. Propensity score matching (1:1) across 24 covariates was performed to balance demographics, comorbidities, medications, and baseline clinical characteristics. Outcomes assessed from 90 days blanking period to 3 years post-ablation included atrial fibrillation recurrence, heart failure exacerbation events, and all-cause hospitalization using risk, survival, and incidence analyses. **Results:** After matching, 678 patients were included in each cohort with well-balanced baseline characteristics. Atrial fibrillation recurrence did not differ significantly between early and delayed ablation groups (hazard ratio [HR] 0.71, 95% CI 0.39–1.28). In contrast, delayed ablation (>1 year) was associated with a significantly higher risk of heart failure events (HR 0.62, 95% CI 0.54–0.70) and shorter median heart failure-free survival compared with early ablation. All-cause hospitalization risk was similar between groups, although time-to-event analyses favored ablation within 1 year (HR 0.71, 95% CI 0.59–0.85). **Conclusions:** In this large, real-world cohort of older patients with atrial fibrillation and systolic heart failure, late ablation was associated with worse 3-years heart failure outcomes and delayed hospitalization-free survival, despite similar atrial fibrillation recurrence rates. These findings suggest that ablation timing may differentially influence heart failure progression independent of rhythm recurrence and highlight the need for prospective studies to define patient-specific timing strategies in elderly populations.

## 1. Introduction

Atrial fibrillation (AF) and heart failure with reduced ejection fraction (HFrEF), commonly occur together, forming a mutually reinforcing interplay that significantly worsens prognosis [1,2]. AF can exacerbate heart failure through loss of atrial contraction, irregular ventricular response, and tachycardia-induced cardiomyopathy, while underlying ventricular dysfunction promotes atrial remodeling and the development of AF. When present concurrently, these conditions are associated with substantially higher risks of mortality and hospitalization than when either disorder occurs in isolation [3].

Catheter ablation has become a well-validated and increasingly preferred rhythm-control strategy for AF in patients with HFrEF, a conclusion reinforced by multiple randomized trials demonstrating its superiority over medical therapy in improving arrhythmia outcomes and clinical status [4,5,6]. As a result, catheter ablation has become an increasingly recommended treatment option in contemporary clinical practice guidelines.

Despite the growing body of evidence supporting catheter ablation in this population, important questions remain regarding the optimal timing of intervention, particularly among older adults (≥60 years old). Prior studies have suggested that earlier rhythm control strategies may be associated with improved outcomes in AF; however, most randomized trials evaluating catheter ablation enrolled relatively younger populations and did not specifically examine the impact of ablation timing in elderly patients with HFrEF. Consequently, current guidelines acknowledge the potential importance of earlier intervention but do not provide specific recommendations regarding the timing of ablation in this high-risk subgroup [4].

Given the rising incidence of AF and HFrEF in aging populations, coupled with the expanding use of catheter ablation and the lack of prospective studies specifically evaluating the optimal timing of this intervention in elderly patients, real-world evidence has become essential to inform individualized, age-appropriate treatment strategies [4,7]. Accordingly, real-world data may provide valuable insight into treatment patterns and outcomes in patient populations that are often underrepresented in randomized trials.

Therefore, the objective of this study was to evaluate the association between early versus delayed catheter ablation and clinical outcomes in elderly patients with AF and HFrEF using a large real-world database.

## 2. Methods

A retrospective study was conducted using the TriNetX Research Network, a federated platform of de-identified electronic health records from approximately 111 U.S. healthcare organizations (HCOs). The database provides longitudinal patient-level data, including demographics, diagnoses, procedures, medication exposures, and laboratory results, with analyses performed locally at participating institutions and only aggregate, de-identified outputs returned. The study followed the Strengthening the Reporting of Observational Studies in Epidemiology (STROBE) Guidelines and TriNetX publication standards, and all clinical codes used to define cohorts, covariates, and outcomes are provided in the Supplemental Material (Table S1).

### 2.1. Study Cohorts

Adult patients aged ≥ 60 years with HFrEF and AF were eligible for inclusion. Because TriNetX classifies heart failure (HF) using ICD-10 terminology, systolic HF codes were used as a surrogate for HFrEF in this study; this approach has been previously validated, demonstrating that ICD-10 coding for systolic versus diastolic HF reliably distinguishes HFrEF from HFpEF when an ejection fraction threshold of 50% is applied [8]. Patients were required to meet all cohort-defining criteria between 1 January 2010 and 1 January 2022. Two cohorts were constructed based on the timing of catheter ablation for AF. The early ablation cohort consisted of patients who underwent pulmonary vein isolation within 1 year of the qualifying AF and HFrEF diagnosis. The delayed ablation cohort included patients who underwent the same procedure ≥1 year after the qualifying diagnoses. Patients with prior exposure to class I or class III antiarrhythmic medications or abnormal electrocardiographic findings prior to cohort entry were excluded to reduce confounding by prior rhythm-control strategies. The index date, time zero, was defined as the date of catheter ablation for both cohorts, and baseline characteristics were assessed prior to this date, with all outcomes evaluated only during the post-ablation follow-up period.

### 2.2. Outcomes

Outcome assessment began 90 days after the index date (blanking period) to allow for procedural recovery and stabilization and continued through 3 years of follow-up. Patients were followed until the occurrence of an outcome or censoring at the last recorded clinical encounter within the TriNetX network. The primary outcome was AF recurrence and was defined using treatment-based markers available within the database, including repeat direct-current cardioversion, repeat catheter ablation, or initiation or escalation of antiarrhythmic drug therapy. Secondary outcomes included HF events and all-cause hospitalization. For analysis of incident outcomes, patients with documentation of the outcome prior to the start of the analysis window were excluded when appropriate to ensure assessment of new-onset events.

### 2.3. Propensity Score Matching

To account for baseline differences between cohorts, 1:1 propensity score matching (PSM) was performed using a greedy nearest-neighbor algorithm with a predefined caliper within the TriNetX platform. Consistently with TriNetX methodology, patient records were randomized prior to matching for mitigation of order-dependent bias. Covariates were selected a priori based on clinical relevance and the prior literature. Twenty-four covariates were used in PSM including demographic variables, cardiovascular comorbidities, device history, medication use, and relevant laboratory measures, including age, sex, race/ethnicity, hypertension, diabetes mellitus, obesity, dyslipidemia, systolic HF, chronic kidney disease, hypothyroidism, obstructive sleep apnea, presence of implantable cardiac devices, antiarrhythmic and cardiovascular medications, and body mass index. The full list of covariates used in the propensity score model is provided in Supplementary Table S1. Balance between cohorts before and after matching was assessed using standardized mean differences (SMDs), with absolute values < 0.10 considered indicative of adequate balance.

### 2.4. Statistical Analysis

All analyses were conducted using the TriNetX Compare Outcomes analytic framework. Baseline characteristics were summarized before and after PSM. Measures of association included risk differences, risk ratios, and odds ratios for binary outcomes. Time-to-event analyses were performed using Kaplan–Meier methods and Cox proportional hazards models to estimate hazard ratios (HRs) with 95% confidence intervals (CIs). Kaplan–Meier curves were generated for all outcomes to visualize cumulative event incidence over time. Analyses were performed both before and after PSM. Statistical significance was defined as a two-sided *p* value < 0.05. For all models, the delayed ablation cohort served as the reference group, such that HRs < 1 indicated lower risk associated with earlier ablation.

### 2.5. Ethics and Institutional Review Board Statement

This study was conducted using de-identified data from the TriNetX research network and was classified as exempt from the institutional review board (IRB) review by the University of Texas Medical Branch IRB. The analysis did not involve direct interaction with human subjects, and all data satisfied de-identification requirements outlined in Section §164.514(a) of the Health Insurance Portability and Accountability Act (HIPAA) Privacy Rule. All procedures and analyses were performed in accordance with applicable ethical and regulatory standards.

## 3. Results

**Figure 1:**
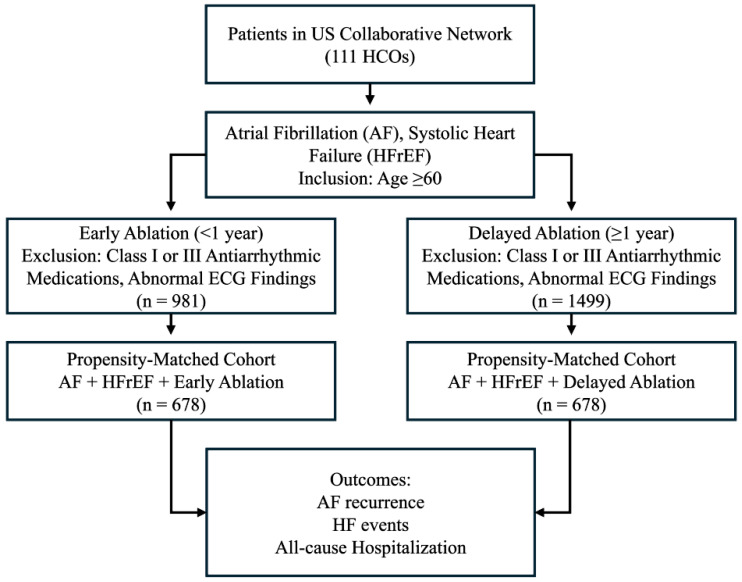
Cohort selection and propensity score-matched design comparing Early versus Delayed Ablation in patients with AF and HFrEF. Abbreviations: AF, atrial fibrillation; ECG, electrocardiogram; HCO, healthcare organization; HF, heart failure; HFrEF, heart failure with reduced ejection fraction.

A total of 2480 patients met eligibility criteria prior to matching, including 1499 who underwent delayed AF ablation and 981 who underwent early ablation. Baseline differences between groups were present before matching. After 1:1 PSM, 678 patients remained in each cohort (Figure 1). Post-matching evaluation demonstrated adequate balance across demographic variables, comorbid conditions, medication exposures, and available laboratory parameters, with standardized mean differences generally below accepted thresholds (Table 1).

After propensity score matching, the median follow-up duration was 778 days in the delayed ablation cohort and 1095 days in the early ablation cohort. In the matched cohorts, AF recurrence occurred in 16.2% of patients in the delayed ablation group and 13.7% of those in the early ablation group (Table 2). Although event rates numerically favored early ablation, time-to-event analysis did not demonstrate a statistically significant difference in recurrence risk between groups (HR 0.71, 95% CI 0.39–1.28). Heart failure events were common in both cohorts but occurred less frequently among patients who underwent early ablation (65.2%) compared with those undergoing delayed ablation (71.8%). Survival analysis demonstrated a significantly lower hazard of HF events associated with early ablation (HR 0.62, 95% CI 0.54–0.70). All-cause hospitalization occurred in 34.8% of the early ablation cohort and 36.0% of the delayed ablation cohort. Despite modest differences in crude event rates, time-to-event analysis demonstrated a significantly lower hazard of hospitalization in patients treated with early ablation (HR 0.71, 95% CI 0.59–0.85).

**Figure 2:**
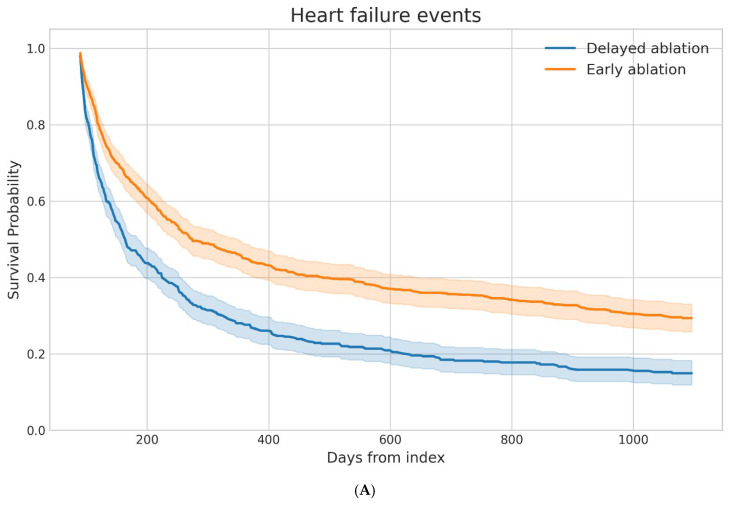

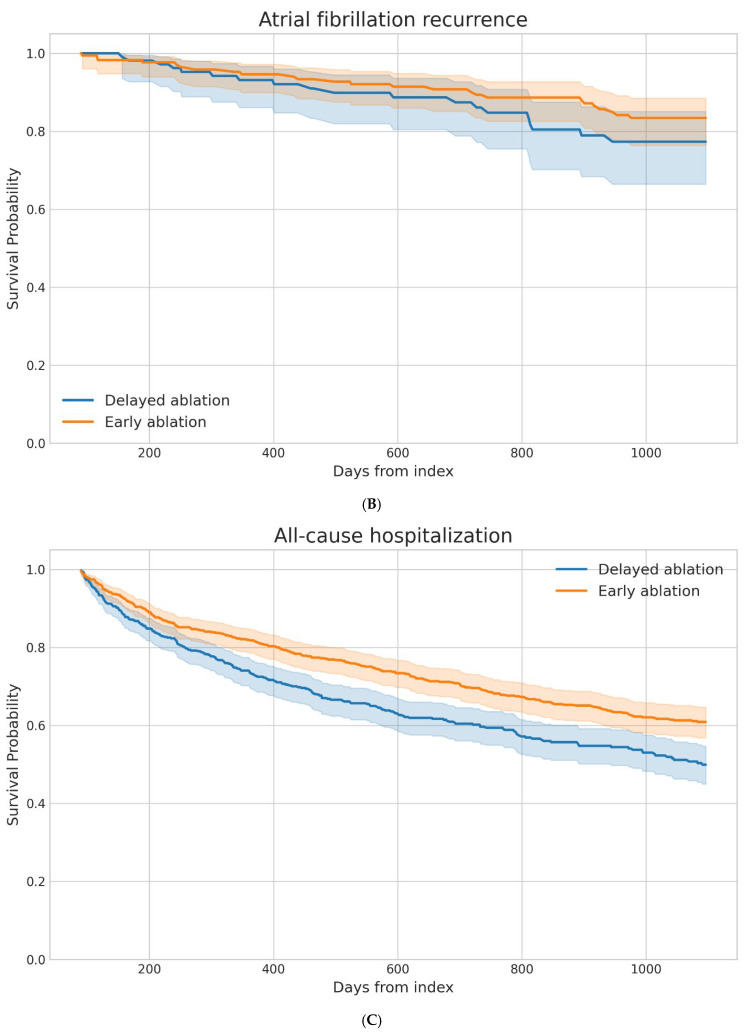
Kaplan–Meier Survival Curves for Clinical Outcomes Following Early Versus Delayed Catheter Ablation. (**A**) Heart failure events, (**B**) atrial fibrillation recurrence, and (**C**) all-cause hospitalization. Shaded areas represent 95% confidence intervals. Time is shown in days from the index procedure.

Kaplan–Meier survival curves demonstrated clear temporal differences between the early and delayed ablation cohorts across several outcomes (Figure 2). For all-cause hospitalization, the delayed ablation cohort exhibited an earlier and more pronounced decline in event-free survival, with progressive separation of the curves throughout follow-up. A similar pattern was observed for HF events, where the delayed ablation group showed a steeper reduction in event-free survival early in the follow-up period that persisted over time. In contrast, atrial fibrillation recurrence demonstrated more modest divergence between cohorts, with largely overlapping survival trajectories during much of the observation period.

## 4. Discussion

Catheter ablation timing in patients with AF and HFrEF remains an area of clinical uncertainty, particularly in older populations who are often underrepresented in randomized trials. The present study evaluated the association between early versus delayed catheter ablation and clinical outcomes in elderly patients with AF and HFrEF. In this large propensity-matched analysis, earlier AF ablation in adults ≥ 60 years with HFrEF was associated with significantly lower hazards of HF events and all-cause hospitalization compared with delayed ablation, while no significant difference in AF recurrence was observed. These findings suggest that the timing of ablation may influence downstream HF trajectory independent of binary recurrence outcomes and support a disease-modifying role for earlier rhythm control in this population.

The reduction in HF-related outcomes observed with earlier ablation in the present study is consistent with randomized data supporting the clinical benefit of catheter ablation in patients with AF and HF. In CASTLE-AF, catheter ablation in patients with symptomatic AF and reduced ejection fraction significantly reduced the composite of all-cause mortality or HF hospitalization compared with medical therapy and was also associated with lower all-cause mortality [9]. Similarly, in the prespecified HF subgroup of CABANA, ablation was associated with lower mortality and fewer HF hospitalizations compared with drug therapy, supporting a potential benefit of rhythm control in AF-HF populations [10]. Although RAFT-AF did not demonstrate a statistically significant reduction in its primary composite endpoint of death or HF events with ablation-based rhythm control compared with rate control, outcomes were numerically lower and several secondary measures including left ventricular function, exercise capacity, biomarkers, and quality of life favored ablation [11]. Collectively, these trials established the benefit of ablation relative to medical therapy but did not specifically address whether the timing of ablation after diagnosis influences outcomes. The present findings extend this literature by suggesting that earlier intervention may confer additional clinical benefit even among patients who ultimately undergo ablation.

Timing-dependent benefit has been increasingly recognized across the AF treatment spectrum. The EAST-AFNET 4 trial demonstrated that early rhythm control initiated soon after AF diagnosis reduced cardiovascular outcomes compared with usual care, supporting the concept that earlier intervention during the remodeling window may alter disease progression [12]. Observational data examining diagnosis-to-ablation time likewise suggest that shorter intervals from AF diagnosis to ablation are associated with improved arrhythmia outcomes and, in some analyses, lower hospitalization risk [13]. The present analysis reinforces these timing signals in an older, HF-specific population and suggests that delaying ablation may permit progressive adverse remodeling that becomes less reversible over time.

Notably, earlier ablation was associated with improved HF outcomes despite no significant difference in AF recurrence, underscoring the limitations of recurrence as a binary endpoint and raising the possibility that reductions in AF burden may be more closely linked to clinical benefit. In CAMERA-MRI, post-ablation AF burden was quantified with an implanted loop recorder and catheter ablation led to marked improvement in left ventricular function compared with rate control [14]. Consistent with this paradigm, studies evaluating continuous monitoring metrics suggest that lower AF burden after ablation is associated with more favorable clinical outcomes, supporting AF burden as a mechanistically relevant target beyond ‘any recurrence’ [5,15]. Ablation may therefore improve HF trajectory through reduction in tachycardia-mediated cardiomyopathy, improved atrioventricular synchrony, and attenuation of neurohormonal activation, even when intermittent AF persists [16]. Procedural factors may also influence rhythm outcomes after AF ablation. Recent studies suggest that ablation strategies and energy delivery parameters can significantly affect lesion durability and long-term rhythm control, highlighting the evolving nature of ablation techniques and their potential impact on recurrence rates [17].

The present findings also have implications for the management of older adults with AF and HFrEF, a population often referred for ablation later in the disease course due to concerns about procedural risk and comorbidity burden [7]. Prior studies have demonstrated that older patients derive similar symptomatic and functional benefit from ablation as younger cohorts, though referral patterns remain conservative [18]. The observed association between earlier ablation and improved HF outcomes in this analysis suggests that age alone should not justify delaying rhythm control strategies when otherwise clinically appropriate.

From an electrophysiology practice perspective, these data support consideration of earlier referral for catheter ablation in patients with AF and HFrEF, particularly when rhythm control is pursued. While randomized trials comparing early versus delayed ablation in HF populations are limited, the totality of evidence including EAST-AFNET 4 and prior ablation trials suggests that intervention during earlier disease stages may provide the greatest opportunity to modify long-term outcomes [5,9,10,12,13,15,18].

## 5. Limitations

Several limitations should be considered when interpreting these findings. This was a retrospective observational analysis using EHR data, and although PSM was employed to balance measured covariates between groups, residual confounding from unmeasured factors cannot be excluded [19]. Administrative and registry-based datasets lack granular clinical detail, including symptom burden, frailty indices, procedural complexity, operator experience, and variability in data capture across participating healthcare organizations, all of which may influence both timing of ablation and outcomes. In addition, observational studies evaluating AF ablation are subject to referral and treatment-selection biases, whereby patients selected for earlier intervention may differ systematically from those treated later despite statistical adjustment [4,20,21]. AF recurrence was defined using diagnosis codes, repeat procedures, and treatment markers rather than continuous rhythm monitoring. As a result, asymptomatic or subclinical AF episodes may have been under-detected, and recurrence may be underestimated relative to studies using implantable monitors or structured follow-up. Binary recurrence endpoints are increasingly recognized as imperfect surrogates for AF burden, which may more closely relate to clinical outcomes, particularly in patients with HF [5,22,23]. Differences in AF burden reduction between groups may therefore have contributed to observed differences in clinical outcomes despite similar recurrence rates. The dataset lacked detailed echocardiographic and procedural variables, including left ventricular ejection fraction trajectories, atrial size, fibrosis burden, ablation strategy, and procedural success parameters. In addition, procedure identification relied on CPT coding and therefore did not permit differentiation between specific ablation modalities such as radiofrequency, cryoballoon, or pulsed-field ablation. These factors are known to influence both recurrence and HF outcomes after ablation [9]. The absence of continuous rhythm monitoring, imaging, and medication adherence data further limits mechanistic interpretation of the observed associations. Because continuous rhythm monitoring data were not available, asymptomatic or untreated AF episodes may not have been captured, and AF burden could not be quantified. Consequently, recurrence estimates should be interpreted as treatment-driven markers rather than direct measures of arrhythmia burden.

Coding-based definitions of HF subtype and clinical outcomes may introduce misclassification bias. Although ICD-based identification of systolic HF has been validated in large datasets, misclassification between HF phenotypes and between incident and prevalent events remains possible [8,24]. Similarly, outcome ascertainment relied on coded encounters and may not capture events occurring outside participating health systems.

The timing of ablation relative to diagnosis was used as a surrogate for disease duration and treatment strategy. This approach cannot fully account for variations in clinical decision-making, patient preference, or disease severity at presentation. Patients undergoing delayed ablation may have had longer-standing AF, more advanced atrial remodeling, or differing comorbidity profiles that were not fully captured in the dataset. While propensity matching improves balance across measured variables, it cannot eliminate unmeasured confounding inherent to retrospective analyses.

Prospective studies and randomized trials specifically evaluating the optimal timing of catheter ablation in elderly patients with AF and HFrEF are needed to confirm these findings and better define patient selection and treatment strategies.

## 6. Conclusions

The optimal timing of catheter ablation in patients with AF and HFrEF remains uncertain, particularly among older adults who are frequently underrepresented in randomized trials. In this real-world, retrospective cohort study, earlier catheter ablation in older adults with AF and HFrEF was associated with lower hazards of HF events and hospitalization compared with delayed ablation, without a significant difference in AF recurrence. These findings suggest that the timing of ablation may influence clinical outcomes in this high-risk population.

## Figures and Tables

**Table 1:** Baseline Characteristics of Patients Undergoing Early vs. Late Atrial Fibrillation Ablation Before and After PSM.

Covariate	Before PSM				After PSM			
	Early Ablation	Delayed Ablation	SMD	*p*-Value	Early Ablation	Delayed Ablation	SMD	*p*-Value
Demographics								
Age at index (mean ± SD)	69.3 ± 5.8	72.0 ± 5.8	0.450	<0.001	70.7 ± 5.9	70.4 ± 5.5	0.049	0.366
Female sex (%)	30.4%	29.8%	0.012	0.767	29.1%	28.9%	0.003	0.952
White race (%)	79.6%	81.8%	0.055	0.178	79.6%	79.9%	0.007	0.892
Black or African American (%)	10.1%	11.3%	0.040	0.328	12.1%	11.7%	0.014	0.801
Asian (%)	2.2%	1.9%	0.026	0.516	2.1%	2.4%	0.020	0.712
Hispanic or Latino (%)	3.7%	3.0%	0.037	0.360	3.1%	3.7%	0.033	0.548
Unknown race (%)	5.1%	2.9%	0.110	0.006	3.8%	3.5%	0.016	0.773
Diagnosis								
Hypertension (%)	73.3%	72.8%	0.012	0.779	74.6%	75.1%	0.010	0.851
T2DM (%)	24.3%	27.8%	0.081	0.049	27.3%	28.2%	0.020	0.716
Overweight/obesity (%)	25.7%	25.4%	0.006	0.880	26.3%	26.8%	0.013	0.806
Dyslipidemia (%)	55.4%	56.3%	0.019	0.640	57.1%	57.2%	0.003	0.956
Systolic HF (%)	80.9%	39.0%	0.946	<0.001	72.4%	70.9%	0.033	0.547
CKD stage 3 (%)	12.1%	16.2%	0.117	0.005	14.5%	14.6%	0.004	0.939
CKD stage 4 (%)	1.9%	2.9%	0.061	0.146	1.9%	2.4%	0.031	0.573
Hypothyroidism (%)	10.9%	12.3%	0.043	0.301	12.1%	10.9%	0.037	0.496
OSA (%)	24.1%	24.1%	0.002	0.958	24.6%	23.3%	0.031	0.567
ICD present (%)	10.0%	13.0%	0.095	0.023	13.0%	12.7%	0.009	0.871
Pacemaker present (%)	5.7%	8.3%	0.103	0.014	7.1%	7.2%	0.006	0.916
Cardiac/vascular implants (%)	24.3%	30.7%	0.144	0.001	29.5%	27.6%	0.042	0.434
Medications								
Beta blockers (%)	74.2%	64.1%	0.220	<0.001	71.4%	71.1%	0.007	0.904
Antiarrhythmics (%)	56.6%	53.4%	0.063	0.125	56.0%	55.6%	0.009	0.870
Insulin (%)	16.0%	13.5%	0.069	0.089	15.9%	16.5%	0.016	0.768
Anthropometric measure								
BMI (mean ± SD)	30.5 ± 6.2	30.9 ± 6.4	0.064	0.194	30.5 ± 6.1	30.8 ± 6.3	0.054	0.404

Early ablation (<1 year) and late ablation (>1 year) defined by duration since AF diagnosis. Values are reported as mean ± SD or *n* (%). Propensity score matching was performed 1:1. Standardized mean differences <0.1 indicate good balance. Abbreviations: BMI, body mass index; CKD, chronic kidney disease; HF, heart failure; ICD, implantable cardioverter–defibrillator; OSA, obstructive sleep apnea; PSM, propensity score matching; SD, standard difference; SMD, standardized mean difference; T2DM, type 2 diabetes mellitus.

**Table 2:** Clinical outcomes after PSM among Patients undergoing Early vs. Late Atrial Fibrillation Ablation.

Outcome	Early Ablation Events/N	Delayed Ablation Events/N	ARD (95% CI)	RR (95% CI)	HR (95% CI)	Log-Rank *p*-Value
AF recurrence	25/182 (13.7%)	19/117 (16.2%)	2.5% (−5.8%, 10.9%)	0.85 (0.49–1.47)	0.71 (0.39–1.28)	0.255
HF events	442/678 (65.2%)	487/678 (71.8%)	6.6% (1.7%, 11.6%)	0.91 (0.84–0.97)	0.62 (0.54–0.70)	<0.001
All-cause hospitalization	236/678 (34.8%)	244/678 (36.0%)	1.2% (−3.9%, 6.3%)	0.97 (0.84–1.11)	0.71 (0.59–0.85)	<0.001

Abbreviations: AF, atrial fibrillation; ARD, absolute risk difference; CI, confidence interval; HF, heart failure; HR, hazard ratio; RR, risk ratio.

## Data Availability

All data are publicly available in the TriNetX Database through institutional agreement.

## Supplementary Materials

The following supporting information can be downloaded at: https://www.mdpi.com/article/10.3390/jcm15062191/s1. Table S1: ICD-10, CPT, RxNorm, and laboratory covariates used for cohort definition, propensity score matching, and outcome assessment.
